# Extraction of Sulfathiazole from Urine Samples Using Biosynthesized Magnetic Nanoparticles

**Published:** 2017

**Authors:** Mehdi Maham, Rouhollah Karami-Osboo

**Affiliations:** a *Department of Chemistry, Aliabad Katoul Branch, Islamic Azad University, Aliabad Katoul, Iran.*; b *Mycotoxins Research Laboratory, Iranian Research Institute of Plant Protection, Agricultural Research Education and Extension Organization (AREEO), Tehran, Iran.*

**Keywords:** Extraction, Biosynthesis, Magnetic nanoparticles, Urine, HPLC-UV

## Abstract

The application of Pd/Fe_3_O_4_ nanoparticles (NPs) for the adsorption of sulfathiazole (STZ) from urine samples prior to high performance liquid chromatography-ultraviolet detection was studied. Pd/Fe_3_O_4_ NPs were synthesized using plant extract. Possible impact parameters in the extraction process such as magnetic adsorbents amount, extraction time, sample pH, and desorption conditions were investigated and optimized. Under the optimum conditions, the detection and quantification limits were 10 and 30 ng mL^−1^, respectively. The relative standard deviation for five measurements of 100 ng mL^-1^ of STZ was 5.8 %. The proposed method was used for the analysis of different urine samples, and acceptable recoveries in the range of 87.6 – 101.3% were obtained. These results indicated that biosynthesized Pd/Fe_3_O_4_ NPs can be used as an efﬁcient adsorbent for extraction of sulfathiazole from urine samples.

## Introduction

Sulfonamides are a large group of synthetic antibiotics and are consumed worldwide in veterinary medicine (1); and also, they are used as additives in animal feed (2). Appropriate withdrawal time must be observed before slaughtering or milking to avoid contamination of meat and milk obtained from medicated animals (3, 4). This is important because sulfonamides as one of the most common antibiotics residues in animal feed, cause serious health risks to humans, such as the increase of the hazard of developing antibiotic resistance and lead to allergic or toxic effects (5, 6). Sulfonamides in the human body can be excreted unchanged via urine (7). Furthermore, collect of urine samples are easier than other biological materials, such as blood or tissue. Thus, determination of sulfonamides in human urine is a good way for toxicological and clinical chemistry studies.

**Figure 1:**
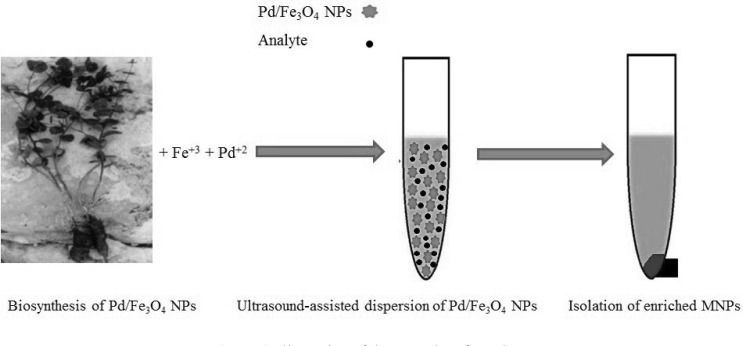
Illustration of the procedure for MSPE**.**

**Figure 2:**
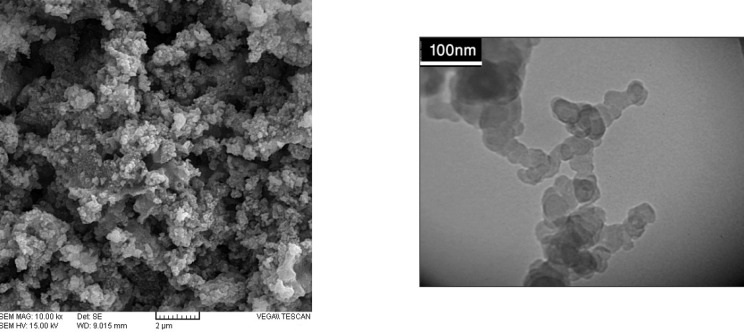
**A) **SEM image of Pd/Fe_3_O_4_ NPs. **B) **TEM image of Pd/Fe_3_O_4_ NPs

**Figure 3:**
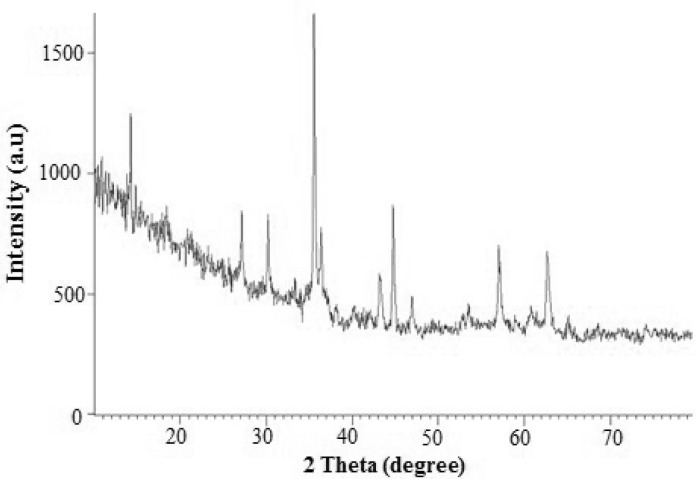
XRD powder pattern of the Pd/Fe_3_O_4_ NPs.

**Figure 4:**
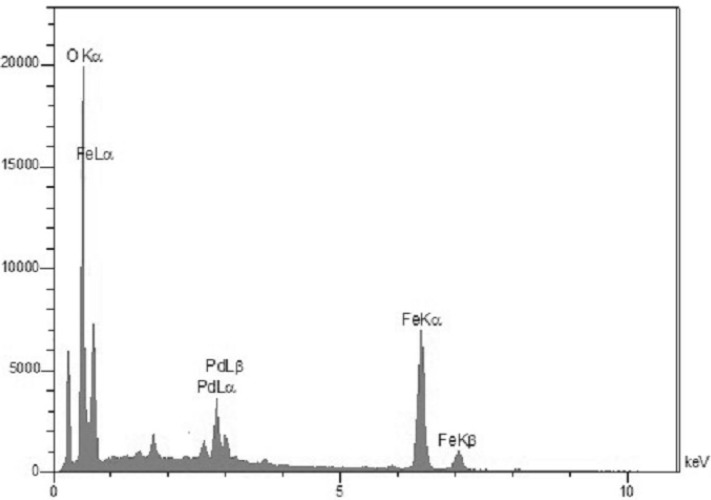
EDS spectrum of Pd/Fe_3_O_4_ NPs.

**Figure 5:**
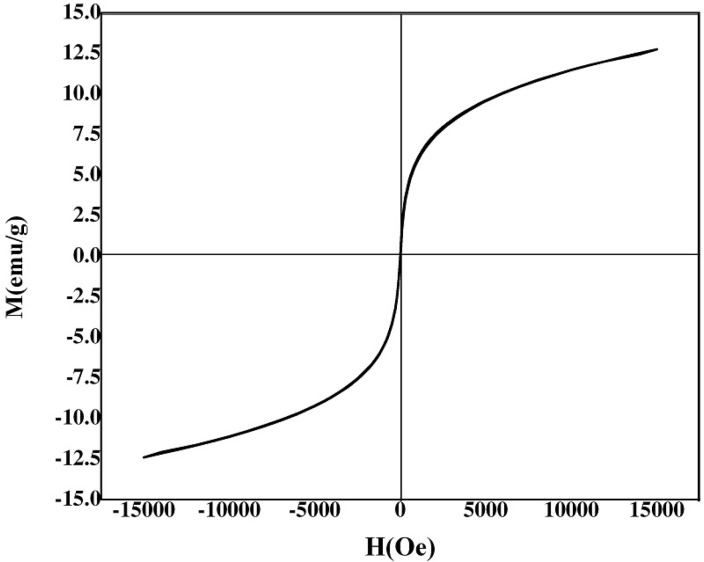
VSM magnetization curve of Pd/Fe_3_O_4_ NPs.

**Figure 6:**
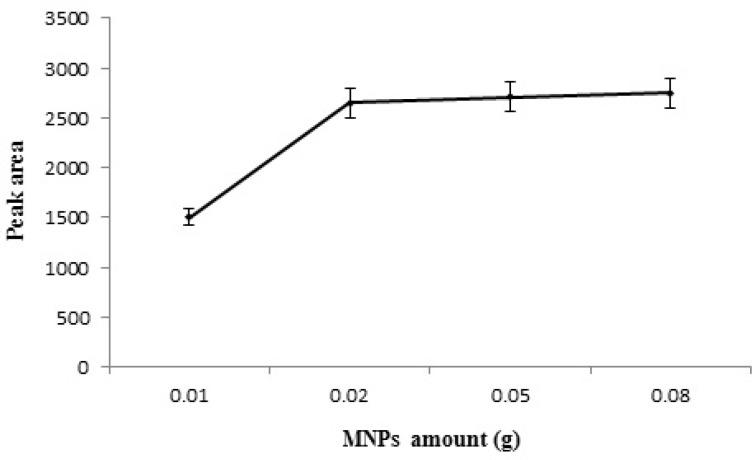
Effect of Pd/Fe_3_O_4_ NPs amount on the extraction efficiency of STZ.

**Figure 7:**
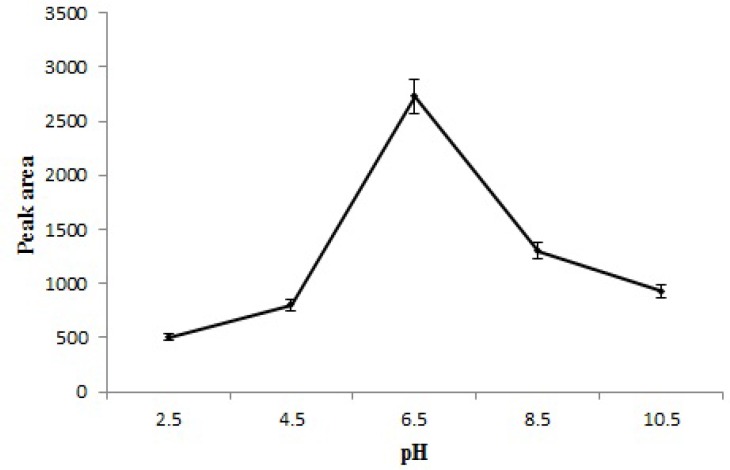
Effect of pH on the extraction efficiency of STZ.

**Figure 8:**
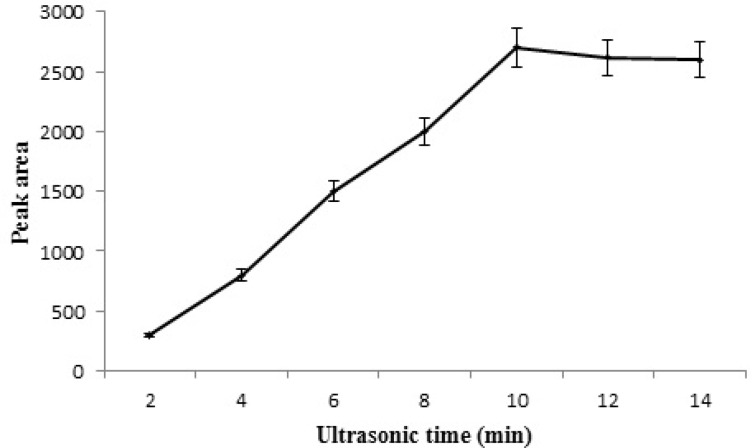
Effect of extraction time on the extraction efficiency of STZ.

**Figure 9:**
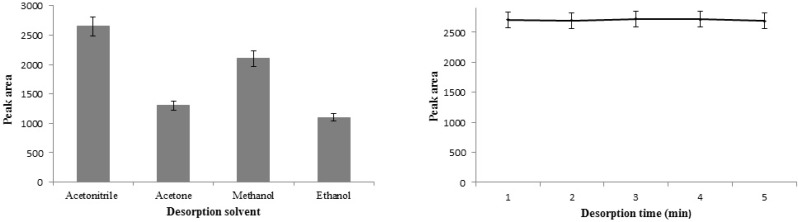
Desorption conditions: **A) **Desorption solvent. **B) **Desorption time.

**Table 1:** Relative recoveries of STZ in urine samples^a^.

**Analyte**	**Number samples**	**Initial concentration** **mean ± SD**^b^ **(ng mL-1)**	**Concentration added (ng mL** **-1** **)**	**Concentration determined mean ± SD (ng mL** **-1** **)**	**Relative recovery** **(%)**
STZ	1	NDc	50.0	48.4 ± 2.4	96.8
			100.0	90.4 ± 4.6	90.4
			200.0	202.6 ± 12.1	101.3
	2	ND	50.0	44.9 ± 2.8	89.8
			100.0	95.2 ± 5.8	95.2
			200.0	188.6 ± 11.1	94.3
	3	ND	50.0	46.15 ± 2.5	92.3
			100.0	91.2 ± 6.2	91.2
			200.0	175.2 ± 9.9	87.6

a Extraction conditions: the amount of MNPs: 0.02 g; the extraction time; 10 min; desorption solvent: 4 mL acetonitrile;

b SD: Standard deviation (n=3).

c ND: not detection

**Table 2:** Comparison of MSPE-HPLC-UV with other methods for the extraction and determination STZ

**Extraction method**	**Detection method**	**LOD (ng mL** **-1** **)**	**RSD (%)**	**Ref.**
HFRLM	HPLC–MS/MS	8.7	< 20	(27)
LLE-FPVLT	HPLC–MS/MS	11.28	>12.9	(28)
SPE	nano-HPLC-MS	8	9	(29)
SLM	HPLC-MS	< 20	-	(30)
MSPE	HPLC-UV	10	5.8	This work

HFRLM, hollow fiber renewal liquid membrane

HPLC–MS/MS, high performance liquid chromatography–tandem mass spectrometry LLE-FPVLT, liquid–liquid extraction with fast partition at very low temperature

SPE, solid-phase extraction

SLM, supported liquid membrane.

Monitoring of sulfonamides at trace and sub-trace level in urine samples requires the use of sensitive and reliable pre-concentration procedure before instrumental analysis to eliminate the matrix interference and enrich the trace levels of sulfonamides residues. Several methodologies have been reported for extraction and pre-concentration of pharmaceutical compounds (8-11). Traditional pre-treatment methods have several disadvantages such as requiring large amounts of toxic organic solvents. So, they are relatively expensive, tedious and time consuming. Nowadays nanoscience has provided a new fascinating insight into the modern world. Nanometer-sized adsorbents have attracted considerable interest owing to their special properties (12, 13). Magnetic nanoparticles (MNPs) as the adsorbents have been effectively used in the extraction and removal of some organic and inorganic compounds (14-17). The MNPs are super paramagnetic and can be easily tracked, manipulated and separated under external magnetic ﬁelds. Magnetic solid phase extraction (MSPE) can be accomplished without the need of centrifuging or ﬁltering, which cause simpler and faster separation. Despite the many benefits of using MNPs but pure magnetic particles have some inherent limitations such as the tendency to form aggregates and changing the magnetic properties in complex environmental and biological samples. There are several studies based on using protective coating on magnetic particles to avoid inherent limitations of these adsorbents (14-16). But most of them have relatively tedious synthetic procedures and use toxic and expensive chemicals. 

Green synthesis of NPs by using various biological systems such as bacteria, fungus, plant and fruit extracts is highly desirable (18-20). In continuation of our recent works on the green chemistry and application of NPs (18-22), we used biosynthesized Pd/Fe_3_O_4_ NPs as adsorbent in the extraction of sulfathiazole (STZ) from urine samples. Pd/Fe_3_O_4_ NPs were synthesized by utilizing *Euphorbia condylocarpa M. bieb* root extract. *Euphorbia condylocarpa* is a plant species in the genus Euphorbia. Different parts of *Euphorbia condylocarpa* contain phytochemicals such as ﬂavonoids (FIOH), tetracyclic triterpenoids, and trifolin (23, 24). Biosynthesis of NPs has several advantages over traditional synthetic methods such as simple reaction setup, low expenditure, and eliminates dangerous as well as toxic chemicals. MSPE was done based on ultrasonic action and the enriched adsorbents were separated from the matrix rapidly with a strong magnetic field.

## Experimental

*Reagents and materials*

The HPLC-grade acetonitrile, methanol, and all analytical grade extraction solvents were purchased from Merck (Darmstadt, Germany). Deionized water was obtained from a Mili-Q- milipore (Milford, MA, USA). Analytical standard (pestanal quality) of STZ was purchased from Sigma–Aldrich (Germany). A Stock solution of STZ (1000 µg mL^-1^) was prepared by dissolving 10 mg in 10 mL methanol and stored at -20 ˚C. The working standard solutions were prepared by successive dilution of the stock solution with methanol. Drug free urine samples were collected from adult volunteers. 

*Apparatus*

The liquid chromatograph (Waters, USA) equipped with auto sampler (waters 717), binary HPLC pump (Waters 1525) and a dual λ absorbance UV detector (Waters 2487). The HPLC separation was performed on a chromolith HPLC column (15 cm, Merck) at the column temperature of 35 ^o^C. A mixture of water and acetonitrile (9:1 v/v) were used as mobile phase and the ﬂow rate was held at 1.2 mL min^-1^ in isocratic elution mode and the eluent was monitored at 270 nm. Surface morphology and composition of biosynthesized Pd/Fe_3_O_4_ NPs were investigated by scanning electron microscopy (SEM) (Philips XI30) equipped with an energy dispersive X-ray spectroscopy (EDS) (Philips D6792). The size of MNPs was studied by transmission electron microscopy (TEM) (Zeiss-EM10C-80 KV). X-ray diffraction (XRD) measurements were carried out using a Brucker D8 Discover (Cu Kα = 1.5406 Å). The scanning rate was 2^o^/min in the 2θ range from 10 to 80^o^. A 40 KHz and 0.138 KW ultrasonic water bath with temperature control (Tecno-Gaz SpA, Italy) was used for ultrasound-assisted magnetic solid phase extraction. A pH meter model 713 (Metrohm, Swiss) and vortex (IKA, USA) were used for adjustment of the pH of solution and desorption of STZ, respectively.

*Preparation of Euphorbia condylocarpa M. bieb root extract*

Dried root powder of *Euphorbia condylocarpa* M. bieb (50 g) was extracted by boiling in double distilled water (300 mL, for 20 min). Then the aqueous extract was centrifuged (7000 rpm) and the obtained supernatant was used as extract.

*Synthesis of Pd/Fe*_3_ *O*_4_ *NPs*

Pd/Fe_3_O_4_ NPs were synthesized by using *Euphorbia condylocarpa* M. bieb root extract as reducing agents and stabilizers. Firstly, 0.5 g FeCl_3_.6H_2_O and 0.1 g PdCl_2_ were dissolved in 30 mL aqueous extract of the *Euphorbia condylocarpa* while the solution was stirring strongly (at 60^ o^C). Then, a solution of 1.0 M Na_2_CO_3_ was added dropwise to the mixture to obtain alkaline pH (during 35 min). Suspension of MNPs was formed by stirring (for 5 h, at 60 °C). Precipitate of Pd/Fe_3_O_4_ NPs was obtained by centrifuging the suspension at 7000 rpm and then MNPs were dried after washing the sediment with ethanol and distilled water, respectively.

*MSPE procedure*

The MSPE procedure was carried out as shown in Figure 1. Firstly, 0.02 g Pd/Fe_3_O_4_ NPs were added into 10 mL spiked sample (100 ng mL^-1^). The mixture was ultrasonicated for 10 min. Then, the enriched NPs were separated from solution by using a strong magnet and the supernatant was discarded. The target analyte was desorbed by 4 mL acetonitrile (2 mL for two times) by vortexing for 1 min. The eluate was combined and after evaporation of the solvents, the residue was dissolved in 1000 µL mobile phase. The aliquot of 100 µL final solution was used for further liquid chromatography.

## Results and Discussion

*Characterization of Pd/Fe*_3_ *O*_4_ *NPs*

The surface morphology and size of Pd/Fe_3_O_4_ NPs were studied by SEM and TEM (Fig. 2). The SEM image revealed that the surface of Fe_3_O_4_ was modiﬁed with Pd (Figure 2A). Bright dots over the surface of iron oxide are related to Pd NPs. The TEM images showed that the size of Pd/Fe_3_O_4_ NPs was less than 100 nm (Figure 2B). Phase investigation of the magnetic adsorbent was performed by XRD and the powder di-raction pattern of the Pd/Fe_3_O_4_ NPs is presented in Figure 3. The presence of iron and palladium was confirmed by powder XRD measurements. The elemental composition of the Pd/Fe_3_O_4_ NPs was investigated by EDS. This analysis established that the MNPs were composed of Fe, Pd and oxygen (Figure 4). Vibrating sample magnetometer (VSM) magnetization curve shows that Pd/Fe_3_O_4_ NPs exhibit typical superparamagnetic behavior due to not exhibiting hysteresis (Figure 5). 

*Reduction of palladium ions*

The mechanism for the formation of Pd NPs can be described as shown in Scheme 1. Pd ions were reduced to nano zero valent (NZV) metallic particles using FlOH contents of the plant as a green reducing agent which can be replaced with commonly used hazardous reducing agent.

nFIOH + Pd^+2^ → nFIO (Radical) + nPd^0^

nFIO (Radical) + Pd^+2^ → nfIOX + nPd^0^ (Nucleation)

nPd^0 ^+ Pd^+2^ → Pd_n_^+2^ (Growth)

Pd_n_^+2^ + Pd_n_^+2^ → Pd_2n_^+2n^

(Pd_2n_^+2n^)n + (FIOH) → Palladium NZV

*Optimization of MSPE conditions*

MSPE combined with LC–UV has been developed for determination of STZ in urine samples. In order to obtain a high MSPE efficiency, several parameters affecting the extraction performance, such as amounts of adsorbent, extraction time, pH of the sample, and desorption conditions were investigated and optimized.

*Effect of Pd/Fe*_3_ *O*_4_ *NPs amount*

NPs offer a noticeable higher surface ratio as compared with the common sorbents (micro-sized). Thus, fewer amounts of NPs are needed to obtain acceptable results. Pd/Fe_3_O_4 _NPs were added in the range of 0.01-0.08 g. According to the experimental data (Figure 6) the extraction efficiency of STZ increased by increasing the amount of MNPs to 0.02 g due to the increase in contact surface of adsorbent with STZ and the greater availability of the adsorbent. When the amount of MNPs was more than 0.02 g, the efficiency of extraction of STZ reached the maximum and then kept constant. So, 0.02 g MNPs was used as optimum amount.

*Effect of solution pH*

Solution pH is one of the most important factors which can influence the extraction efficiency because of its effects on surface binding-sites of the adsorbent and aqueous chemistry. In this study, the pH of the sample solution was varied between 2.5-10.5. The results (Figure 7) showed the highest extraction efficiency of STZ at 6.5. This is related to that a change in pH of the solution leads to different ionic form of STZ and different surface charge of MNPs. Below zeta potential of Fe_3_O_4_ (pH_zpc_ = 6.5) (25), the surface of adsorbent has positive charges and STZ forms cationic species (26). As a result, the electrostatic repulsion between STZ^+^ and the positively charged Fe_3_O_4_ causes ineffective adsorption. Also, when pH is higher than pH_zpc_ of Fe_3_O_4_, the surface of Fe_3_O_4_ is negative and has weak interaction with STZ^¯^. Since the best adsorption can be achieved around pH_zpc_ of Fe_3_O_4, _where STZ is neutral (STZ^0^) and pH of sample solution was about 6.5 so, further experiments were done without changing the pH. 

*Effect of extraction time*

Sufficient contact time is needed to achieve the adsorption equilibrium during extraction. The effect of ultrasonic time on the adsorption of STZ was investigated in the range from 2–14 min. Based on the obtained results (Figure 8) the extraction efficiency first increased as sonication time was extended from 2 to 10 min and then remained almost constant by further increasing of sonication time. 

This was attributed to that the adsorption was a dynamic equilibration process and when the equilibrium was obtained further increase in extraction time didn’t have a significant effect on extraction efficiency. Therefore, 10 min sonication time was used for ultrasonic-assisted MSPE.

*Desorption conditions*

In order to obtain higher extraction efficiency, it is required to select an effective desorption solvent. So, the most commonly used organic solvents such as acetonitrile, methanol, acetone, and ethanol were examined as the elution to desorb the STZ from adsorbent. Based on the obtained data (Figure 9A) acetonitrile was selected because of the best eluting power. Also, the effect of vortex time on the desorption of STZ was investigated between 1 and 5 min. The results (Figure 9B) showed that 1 min desorption time was sufficient for quantitative recovery because of the efﬁcient and rapid desorption process.

*Analytical ﬁgures of merit and validation of the MSPE-HPLC method*

Calibration curve was obtained using standard solutions of STZ under the optimized conditions through external standard method. The precision of the method was expressed in term of the relative standard deviation (RSD) and calculated 5.8 % by analysis of ﬁve samples spiked with 100 ng mL^-1^ of STZ. The limit of detection (LOD) (S/N = 3) and limit of quantification (LOQ) (S/N = 10), were 10 and 30 ng mL^-1^, respectively. To evaluate efficacy of the biosynthesized MNPs in biological samples, three urine samples were collected from apparently healthy volunteers. 1.0 mL of the each drug free urine sample was diluted to 10.0 mL with deionized water to decrease matrix effects and MSPE procedure was done under the optimized conditions. Since none of the STZ was found in the samples, each urine sample was spiked with STZ standard at three concentration levels. After the MSPE procedure, the recovery was calculated by using the corresponding calibration curve to validate the accuracy of the proposed procedure. The good ﬁgures of merit and recoveries (Table 1) demonstrated that the proposed procedure was a precise and reliable method for the analysis of STZ in urine samples.

*Comparison of MSPE-HPLC-UV with other methods*

This proposed MSPE-HPLC-UV technique was compared with other published methods. The respective LOD and RSD of each method are summarized in Table 2. The superiority of the proposed method is related to its Figures of merit such as LOD and RSD, which are comparable to or even better than the ones that obtained with other techniques and acquired without using any complex and expensive instrument or laborious and high cost pre-concentration steps. The results reveal that MNPs based on biosynthesis can be used as an efﬁcient adsorbent for sensitive and reproducible analysis of STZ from urine samples.

## Conclusions

In this study, we have successfully used Pd/Fe_3_O_4_ NPs synthesized with plant extract in the adsorption of STZ from urine samples. Ultrasound-assisted MSPE was accomplished and MNPs were separated by using an external magnetic field. Large surface area of adsorbent and extraction without needing additional centrifugation or filtration resulted in faster, easier, and more effective extraction process. Based on the obtained results the suggested method described great analytical potential to monitor low concentrations of STZ in biological matrix and opened new dimensions to research and development in future studies.
